# Genome analysis reveals a biased distribution of virulence and antibiotic resistance genes in the genus *Enterococcus* and an abundance of safe species

**DOI:** 10.1128/aem.00415-25

**Published:** 2025-04-09

**Authors:** Belay Tilahun Tadesse, Shuangqing Zhao, Liuyan Gu, Carsten Jers, Ivan Mijakovic, Christian Solem

**Affiliations:** 1 National Food Institute, Technical University of Denmark 114320, Kongens Lyngby, Denmark; 2 Novo Nordisk Foundation Center for Biosustainability 587234 , Kongens Lyngby, Denmark; 3 Systems and Synthetic Biology Division, Chalmers University of Technology 11248, Gothenburg, Sweden; Universita degli Studi di Napoli Federico II, Portici, Italy

**Keywords:** *Enterococcus*, virulence gene, antibiotic resistance genes, whole-genome analysis

## Abstract

Enterococci are lactic acid bacteria (LAB) that, as their name implies, often are found in the gastrointestinal tract of animals. Like many other gut-dwelling LAB, for example, various lactobacilli, they are frequently found in other niches as well, including plants and fermented foods from all over the world. In fermented foods, they contribute to flavor and other organoleptic properties, help extend shelf life, and some even possess probiotic properties. There are many positive attributes of enterococci; however, they have been overshadowed by the occurrence of antibiotic-resistant and virulent strains, often reported for the two species, *Enterococcus faecalis* and *Enterococcus faecium*. More than 40,000 whole-genome sequences covering 64 *Enterococcus* type species are currently available in the National Center for Biotechnology Information repository. Closer inspection of these sequences revealed that most represent the two gut-dwelling species *E. faecalis* and *E. faecium*. The remaining 62 species, many of which have been isolated from plants, are thus quite underrepresented. Of the latter species, we found that most carried no potential virulence and antibiotic resistance genes, an observation that is aligned with these species predominately occupying other niches. Thus, the culprits found in the *Enterococcus* genus mainly belong to *E. faecalis*, and a biased characterization has resulted in the opinion that enterococci do not belong in food. Since enterococci possess many industrially desirable traits and frequently are found in other niches besides the gut of animals, we suggest that their use as food fermentation microorganisms is reconsidered.

**IMPORTANCE:**

We have retrieved a large number of *Enterococcus* genome sequences from the National Center for Biotechnology Information repository and have scrutinized these for the presence of virulence and antibiotic resistance genes. Our results show that such genes are prevalently found in the two species *Enterococcus faecalis* and *Enterococcus faecium*. Most other species do not harbor any virulence and antibiotic resistance genes and display great potential for use as food fermentation microorganisms or as probiotics. The study contributes to the current debate on enterococci and goes against the mainstream perception of enterococci as potentially dangerous microorganisms that should not be associated with food and health.

## INTRODUCTION

*Enterococcus* is a genus of Gram-positive bacteria comprising over 64 species, for which more than 40,000 genome sequences can be found in the National Center for Biotechnology Information (NCBI) database. Two *Enterococcus* species, *Enterococcus faecalis* and *Enterococcus faecium*, dominate in the gastrointestinal (GI) tract, and together account for approximately 1% of the adult microbiota (1, 2). In addition to being part of the gut microflora, *Enterococcus* species are frequently found in other environments rich in carbohydrates, including plants and in fermented foods (3–5). There, they are found together with other lactic acid bacteria (LAB), such as *lactobacilli*, *streptococci*, and *lactococci* (6, 7).

In dairy foods, enterococci contribute to flavor development (8). They have been found in cheddar cheese in high numbers (9–11), where their presence was linked to higher flavor intensity and accelerated ripening (9). Like other LAB, they prolong the shelf life of fermented foods through their production of lactic acid, and many are able to produce antimicrobial peptides called enterocins, which hamper growth of unwanted microorganisms, including pathogenic ones (12–15). The central metabolism of enterococci is identical to that of *Lactococcus*, and enterococci, in principle, can replace lactococci in food fermentations (9). In many respects, they are superior to lactococci, for example, in terms of capacity for degrading carbohydrates, thermotolerance and general robustness, and antimicrobial properties, and it has been documented that they can speed up flavor development in fermented foods (9).

Some enterococci exhibit probiotic characteristics, including the ability to adhere to intestinal cells and tolerate GI conditions well (14, 16–18). Biofilms formed by enterococci on the gut epithelium can help protect the gut lining, reduce inflammation, and protect against invasion by pathogenic microorganisms (7, 19), a phenomenon that also has been shown to take place for the important gut symbiont *Bacteroides thetaiotaomicron* (19).

Reports on virulence and antibiotic resistance among enterococci have hampered their widespread use in food fermentations, despite their obvious and well-documented potential (20–22). Most of the reports have dealt with the two species *E. faecalis* and *E. faecium* (1, 2, 23, 24). Pathogenic isolates have been implicated in various nosocomial infections, for example, wound infections, endocarditis, and urinary tract infections (25), and some have been reported to be able to transfer antibiotic-resistance genes (26).

What has received less attention is that many other LAB used in food fermentations, for example, strains of *Lactobacillus* and *Lactococcus*, also harbor antibiotic resistance genes and can be pathogenic (27, 28). There are even pathogenic strains among the species used in food fermentations, for example, lactococci have been reported as fish pathogens (29). The genus *Streptococcus*, which contains *Streptococcus thermophilus* used in dairy fermentations, is renowned for its many pathogenic species, including biofilm-forming ones associated with diseases such as chronic obstructive pulmonary disease (6). Another curiosity is the genus name *Enterococcus*. While many other LAB can be found in the fecal microbiota, often in abundance, none of these are named after the niche they occupy, for example, various *Lactobacillus* and *Streptococcus* species (30–32).

The resilience of enterococci to various environmental stresses, such as pH and high temperatures, makes them attractive for use in food fermentations (33, 34), but before they can be applied in food, their safety needs to be assessed thoroughly. In the European Union, the European Food Safety Authority (EFSA) stipulates that strains should be free of virulence and transferable antibiotic resistance genes (35–37). However, the distribution and prevalence of virulence and antibiotic resistance genes vary greatly among different *Enterococcus* species and even among strains within the same species (4).

Recent genomic studies have shed some light on the genetic diversity and distribution of virulence and antibiotic resistance genes within the *Enterococcus* genus. Several strains of *E. faecium*, *E. lactis*, *E. durans*, and *E. mundtii* have been shown to completely lack virulence genes (3, 12, 35, 38). In this study, we explore hundreds of genomes of enterococci stored in the NCBI database, using different bioinformatics tools and search for presence/absence of virulence and antibiotic resistance genes. Based on the overview generated, we discuss whether the current perception of *Enterococcus* as a being pathogenic is reasonable and whether the “Entero” part of the genus name should be reconsidered, as many isolates appear to originate from plants and other niches besides the animal gut.

## MATERIALS AND METHODS

### Genome data retrieved from the NCBI database

**Fig 1:**
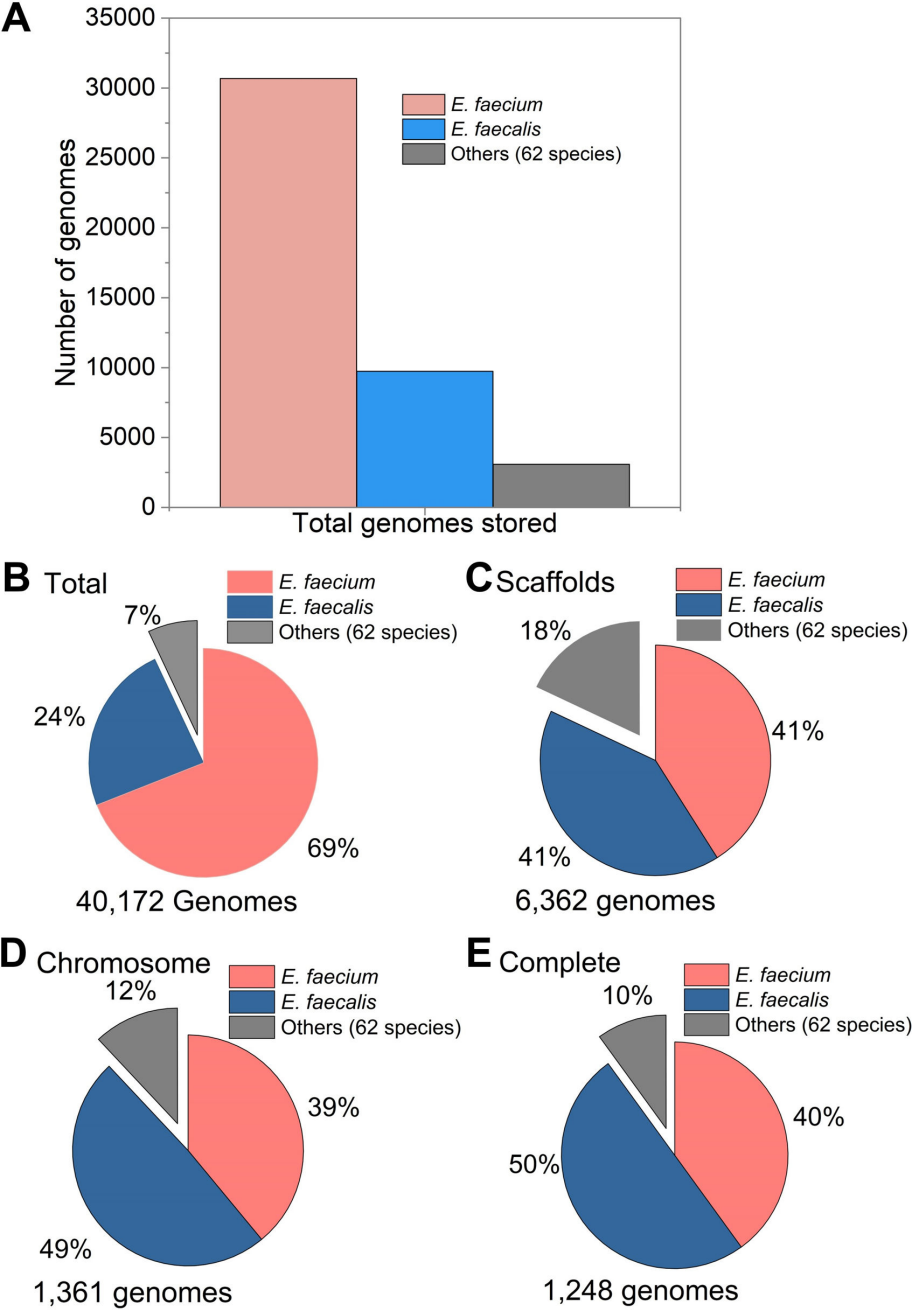
The distribution of *Enterococcus* species based on genome sequences stored in NCBI. Distribution based on total number of genomes (**A**); distribution, in percentage, of total number of genomes (**B**); distribution, in percentage, based on scaffolds (**C**); distribution, in percentage, based on assembled genomes (**D**); and distribution, in percentage, based on assembled and fully annotated genomes (**E**).

To investigate the distribution of virulence and antibiotic resistance genes among *Enterococcus* species, we downloaded the complete genome sequences available in the NCBI database. A total of 1,475 complete genomes were available, whereof 702 annotated with NCBI reference sequences were retrieved. These genome sequences allowed a preliminary assessment of the presence and distribution of virulence and antimicrobial resistance genes. However, most of the complete sequences belonged to the two species *E. faecalis* and *E. faecium*, while the remaining 62 species were represented by fewer genome sequences (Fig. 1E). Therefore, a total of 427 additional genome sequences representing 32 other species that had more than three genome sequences stored in the NCBI database were downloaded and analyzed to get a better understanding of virulence and antibiotic resistance gene distribution in species other than *E. faecalis* and *E. faecium*. Recently, four have been reported to have industrial and probiotic potential. We retrieved sequences corresponding to these species and analyzed them individually: *E. lactis* (200 genomes), *E. durans* (171 genomes), *E. hirae* (170 genomes) and *E. mundtii* (72 genomes). All the sequences analyzed were retrieved in September 2024. The completeness of the genomes was checked by CheckM (Galaxy Version 1.2.3+galaxy0) (39) where genomes with high levels of contamination were excluded.

### Genome annotation and pan-genome-based phylogeny construction

The genomes were annotated uniformly using Prokka (Galaxy Version 1.14.6 + galaxy1) (40, 41) using default settings with similarity, *e*-value cut-off was 1e-06, and a bacterial kingdom genetic code was 11. The core genome was determined using Roary (Galaxy V3.13.0) (42) with default settings, minimum percentage of identity for BlastP was 95%, and percentage of isolates for which a gene to be included in the core genome was 99%. The pan-genome phylogeny tree was produced using the Newick output file of Roary analysis, and the tree was visualized and edited using iTOL V6.

### Virulence factors and antimicrobial resistance screening

All retrieved genomes were analyzed for the presence and absence of virulence factors and antimicrobial resistance genes using ABRicate mass screening of contigs for antimicrobial and virulence genes (Galaxy v.1.0.1) (43) using virulence factor database (44, 45) using default settings, minimum DNA percent of identity was 80%, minimum DNA percentage of coverage was 80%. Resfinder (46) was used with default settings with minimum DNA percentage of identity was 80%, and minimum DNA percent of coverage was 80%.

## RESULTS AND DISCUSSION

### Genome-based analysis of the type strains of genus *Enterococcus*

By the end of September 2024, the NCBI database contained an extensive collection of 40,172 whole-genome sequences for enterococci stored as contigs, scaffolds, chromosomes, and complete genomes. We found that the entries were largely dominated by two species: *E. faecium* and *E. faecalis*, representing 93% of the sequences, while the remaining 7% comprised 62 species (Fig. 1A through E).

**Fig 2:**
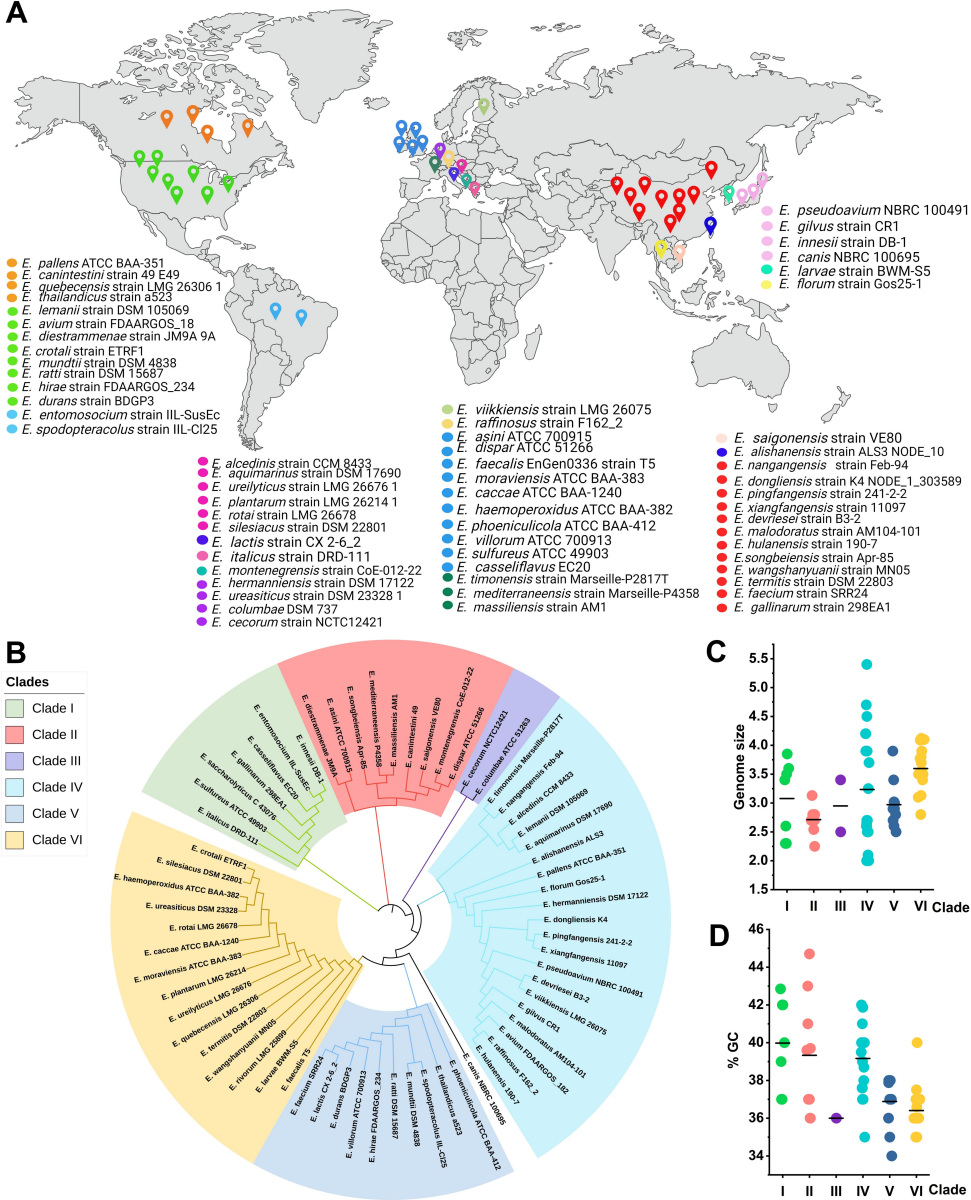
Overview of *Enterococcus* type strains. Origin of type strains indicated on a map based on metadata retrieved from NCBI (**A**). The distinct colors indicate the type strain isolated from the same country. The map was created using BioRender. Phylogenetic tree based on whole-genome sequences for 64 type strains (**B**). The genome size (**C**) and G + C (**D**) content for the 64 *Enterococcus* type strains.

In Fig. 2A, we indicate the origin of the genome sequenced type strains on a map, based on information found in NCBI’s BioProjects. This mapping could potentially provide a better understanding of the environmental and geographic niches occupied by the species and reveal information about their ecological versatility and adaptability. It has been reported that enterococci are widely distributed and can be found in diverse environments across the globe (8).

The taxonomy of *Enterococcus* has evolved significantly, expanding from 35 recognized species in 2014 (47) and 49 in 2017 (48) to currently 64 species (NCBI reference sequence repository; September 2024). Using a genome-based phylogenetic analysis, we clustered the 64 reference species of *Enterococcus* into six distinct clades (a group of organisms believed to have evolved from a common ancestor) (Fig. 2B). One exception was *Enterococcus canis*, which did not fit into any of the six clades. This outlier position suggests that *E. canis* has been subject to a unique evolutionary pressure or genetic development, which could have implications for understanding species-specific adaptation. With the expanding scope of genomic studies carried out on *Enterococcus*, it is expected that the taxonomy most likely will continue to evolve (49).

We found considerable variation in genome size and GC content for the type species analyzed (Fig. 2C and D), which indicates a level of evolutionary diversification among the species. Clades VI and IV were the most diverse clades, containing 15 and 20 species, respectively. Clade IV emerged as particularly diverse, exhibiting the largest genome size variability ranging from 2.0 to 5.4 Mb. This clade includes *Enterococcus timonensis* strain Marseille-P2817T (2.1 Mb), *Enterococcus nangangensis* strain Feb-94 (2.0 Mb), and *Enterococcus pingfangensis* strain 241-2-2 (2.0 Mb), which have the smallest genomes in the clade, and *Enterococcus pallens*, which represents the upper end of the spectrum with a genome size of 5.4 Mb. These differences in genome size show that evolution of *Enterococcus*, like that of other LAB, to a large extent is driven by gene loss, duplication, and acquisition (50). Clade III was less diverse and only contained two species, *Enterococcus cecorum* and *Enterococcus columbae*. In this analysis, we found that Clades I, II, and IV had a relatively high GC content. Generally, the GC content of the genus ranged from 34% to 45%.

### Prevalence of virulence genes

Certain isolates of the two species *E. faecalis* and *E. faecium* have been reported to harbor virulence genes that contribute to their pathogenicity (21, 51). A number of key virulence genes have been reported for *Enterococcus*: *esp*, encoding a surface protein, which is involved in biofilm formation and adherence to host tissues (36, 52); *hylEfm*, found predominantly in *E. faecium*, which encodes hyaluronidase, an enzyme that breaks down hyaluronic acid in host tissues, thereby facilitating bacterial spread (53); *asa1*, which encodes an aggregation substance, which promotes bacterial aggregation and conjugation and enhances transfer of antibiotic resistance genes; *gelE*, which encodes gelatinase, an enzyme that degrades gelatin, collagen, and other host proteins, thus aiding in tissue invasion; and finally the *cyl* cluster, which encodes cytolysin, a toxin that can lyse red blood cells and other host cells (54). Besides these, there are genes encoding pheromones and lipoteichoic acid which are also implicated in virulence of enterococci (51). Although adherence genes are classified as potential virulence genes, these genes are commonly found in different food grade and probiotic strains, for example, in *S. thermophilus* (55, 56) and in approved commercial *Enterococcus* probiotics (2, 57). Adherence is essential for probiotic strains to colonize and persist in the gut (58). EFSA provides clear guidelines for the safety of enterococci for use in animal nutrition: they must not harbor any of the genetic elements IS16, *hylEfm* (hyaluronidase) and *esp*, where the latter encodes an enterococcal surface protein (36, 53).

**Fig 3:**
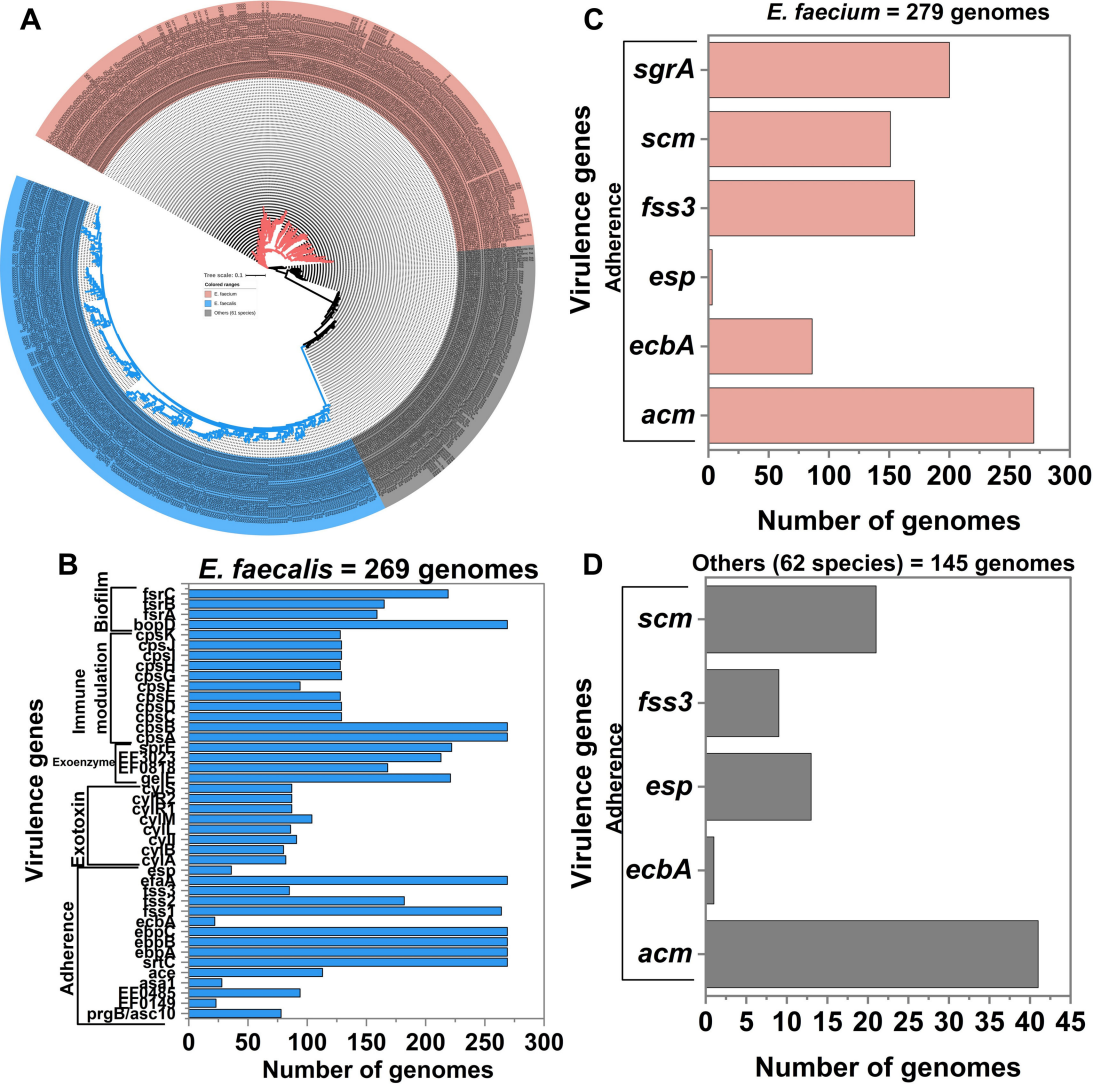
Phylogeny and prevalence of virulence genes. Pangenome-based phylogenetic tree based on 702 annotated *Enterococcus* genome sequences stored in the NCBI database (**A**). Prevalence of virulence genes in the genomes of *E. faecalis* (**B**). Virulence gene prevalence in the genomes of *E. faecium* (**C**). Virulence gene prevalence in the genomes of 62 other species (**D**).

In this analysis, we scrutinized a total of 702 complete *Enterococcus* genomes from the NCBI database, all of which were annotated. The pangenome-based phylogenetic tree (Fig. 3A) revealed that *E. faecalis* had a longer branch length, suggesting significant genetic changes within the species, possibly due to host adaptation and, more recently, due to accumulation of virulence genes. Indeed, our analysis of these genomes revealed a high prevalence of virulence genes. *E. faecalis*, in general, possessed more virulence genes than other *Enterococcus* species (Fig. 3B), including genes encoding proteins that facilitate adhesion, biofilm formation, and evasion of host immune responses. Among the virulence genes found in the genomes of *E. faecalis* were genes needed for synthesizing an immune modulating capsule (*cpsABCDEFGHIK*), the fecal streptococci regulator locus genes (*fsrABC*), cytolysin genes (*cylR2*, *cylL-l*, *cylL-s*, and *cylM*), endocarditis, and genes encoding the biofilm-associated pilus (*ebpABC*). *E. faecalis* is known as the predominant species carrying virulence-associated genes encoding gelatinase (*gelE*), *esp*, and cytolysin (*cylA*), which are all crucial for its pathogenicity.

In contrast, in 279 complete genomes of *E. faecium*, only adherence-related genes could be found. More specifically, the surface-exposed antigen, encoded by *acm*, was the most prevalent virulence gene detected, followed by surface serine-glutamate-repeat-containing-protein A (*sgrA*). Out of the 279 genomes, only 86 contained the gene encoding collagen-binding protein (*ecbA*) from the “Adherence” group of genes. A few genomes contained the *esp* virulence gene (Fig. 3C). The *ecbA* gene is thought to play a role in biofilm development. However, only a limited number of adherence genes have been demonstrated to contribute to biofilm-associated infection *in vivo*. This includes the genes in the *ebpABC* operon, which encodes the endocarditis- and biofilm-associated pilus, and *esp* encoding an enterococcal surface protein (59).

Among the 145 genomes of the remaining 62 species analyzed, the adherence related gene *acm* gene was identified in 41 genomes and the *sgrA* gene in 20 genomes (Fig. 3D). Thus, a comparatively low number of virulence genes is present in *Enterococcus* species other than *E. faecalis* and *E. faecium*. Several genomes did not harbor any virulence genes at all (Fig. S1; Table S1). A high prevalence of virulence genes in *E. faecalis* compared to other species, including *E. faecium*, indicates that *E. faecalis* is the main culprit in the genus. Before the early 1990s, 95% of enterococcal clinical isolates in the hospital setting were *E. faecalis*, and only about 5% were *E. faecium* (37). From the “virulence factor of pathogenic bacteria” home page, it can be seen that most enterococcal infections are caused by *E. faecalis* and *E. faecium*, with other species like *E. durans*, *E. avium*, *E. gallinarum*, and *E. casseliflavus* being less often associated with infections. *E. faecium* is the primary source of vancomycin- and ampicillin-resistant strains, while *E. faecalis* is responsible for 65%–80% of nosocomial enterococcal infections (60).

### Virulence distribution in species other than *E. faecalis* and *E. faecium*

**Fig 4:**
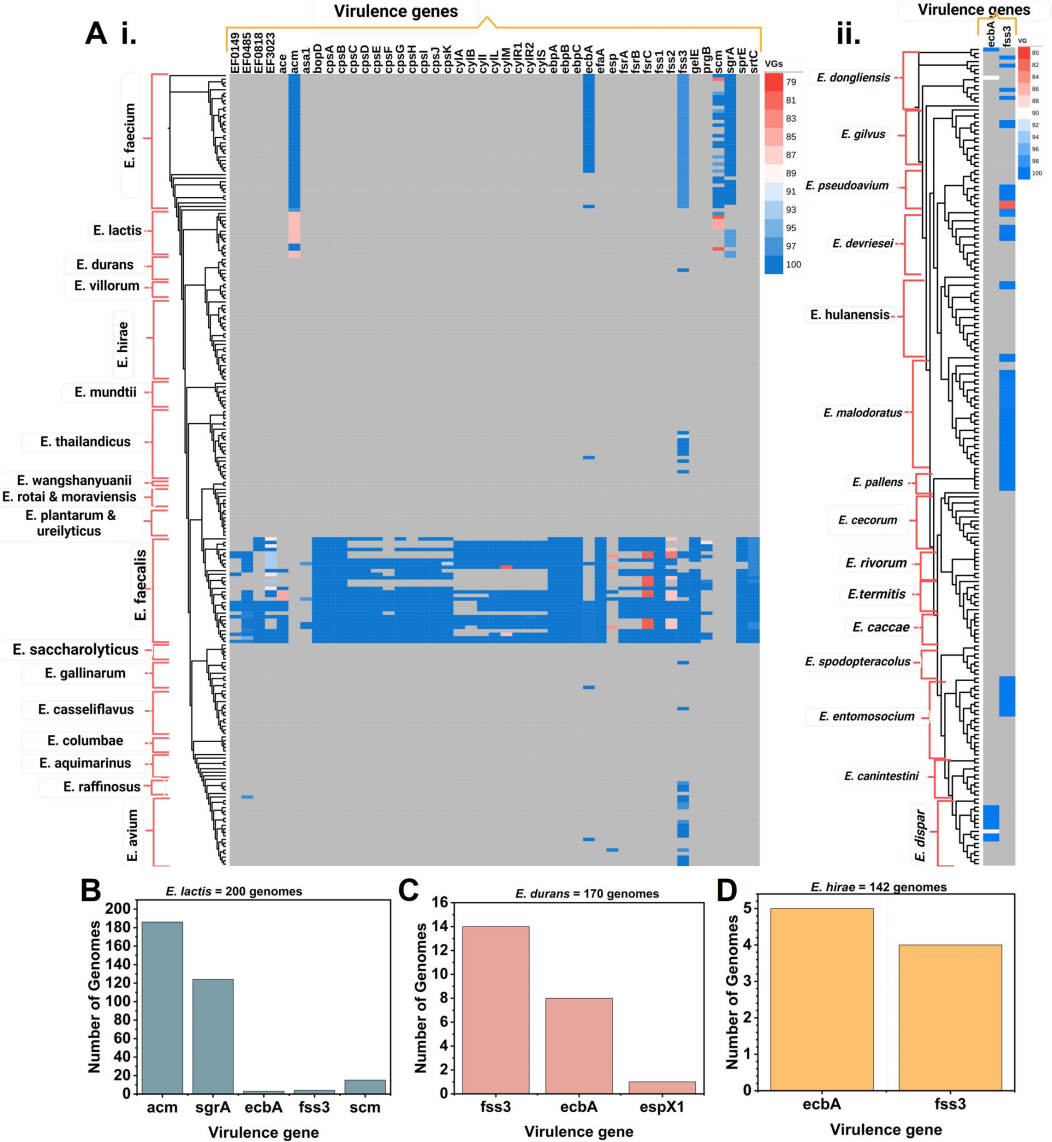
Prevalence of virulence genes in species other than *E. faecalis* and *E. faecium*. The virulence gene distribution in 427 whole-genome sequences of 34 different species downloaded from the NCBI database (**A**). Virulence gene prevalence in the genomes of *E. lactis* (**B**). Virulence gene prevalence in the genomes of *E. durans* (**C**). Virulence gene prevalence in the genomes of *E. hirae* (**D**).

Of the enterococci, only the two species, *E. faecalis* and *E. faecium*, are found in significant numbers in the gut. These two species together account for approximately 1% of the adult human gut microbiota (1, 2). Enterococci have also been found to reside on plants, in soil, and in fermented food products (61–64). When 427 whole-genome sequences representing 32 other species were analyzed, we observed that several species lacked virulence genes in their genomes altogether, including, *E. moraviensis*, *E. aquimarins*, *E. saccharolyticus*, *E. ureilyticus*, *E. plantarum*, *E. rotai*, *E. wangshanyuanii*, *E. mundtii*, *E. pallens*, *E. rivorum*, *E. termitis*, and *E. canintestini* (Fig. 4A, i and ii). Most of these were isolated from plants and some from food (64–68).

Some genomes of *E. lactis* (Fig. 4B), *E. durans* (Fig. 4C), and *E. hirae* (Fig. 4D) contained adherence-related genes (Table S2). For *E. mundtii*, virulence genes could not be detected, except for two genomes that contained a single copy of either *cpsA* or *fss3* (Fig. S2).

The adherence genes identified in the genomes of the *Enterococcus* species besides *E. faecalis* are not considered as virulence determinants (69, 70) and do not facilitate collagen adherence and biofilm formation (71). Shridhar et al. (58) indicated that none of the 22 probiotic strains of *E. faecium* carried major virulence genes required to initiate infections, but many carried genes involved in adhesion to host cells, which may help the strains to colonize and persist in the gut. Among the microbial additives currently authorized, nearly one-third contain strains of *E. faecium*.

Different species within the genus *Enterococcus* are considered food-grade and are widely utilized in fermented food production due to their safety and beneficial attributes (3, 12). It has been reported that several *E. lactis* (35, 72, 73), *E. durans* (74), *E. hirae* (75), and *Enterococcus thailandicus*, which lack virulence and antibiotic resistance genes altogether, have probiotic potential and are suitable as food fermentation microorganisms (38). *E. lactis*, a recently reclassified species, has been recognized as safe and performs well in food fermentation, for example, in dairy products like cheddar and stirred curd cheeses, where it can accelerate ripening (9–11). Creative Biolabs (76) sells different strains of *E. faecium*, *E. lactis*, and *E. durans* that they claim to have probiotic properties. In our previous studies, we have characterized *Enterococcus* species isolated from different vegetables and fermented food products and found several safe species lacking virulence genes (4). Similarly, *E. mundtii* has recently attracted research interest due to its probiotic potential and applications in food and pharmaceuticals (77–80).

#### Prevalence of antibiotic resistance genes

Evidence suggests that the rise of antibiotic resistance in enterococci is largely due to the overuse of antibiotics in agriculture. For instance, avoparcin, commonly used as a growth promoter in pigs and poultry, has led to vancomycin resistance in enterococci (81). Currently, antibiotic-resistant *Enterococcus* species are being reported, where special attention has been given to vancomycin resistance (48). The glycopeptide vancomycin is a first-choice alternative to the penicillin–aminoglycoside combination for treating enterococcal infections, and therefore, the rapid spread of vancomycin-resistant *Enterococcus* (VRE) strains, especially *E. faecalis* and *E. faecium*, has been of particular concern (82). Multiresistant nosocomial isolates have been mentioned to be a threat to immunocompromised and critically ill patients (28, 83).

**Fig 5:**
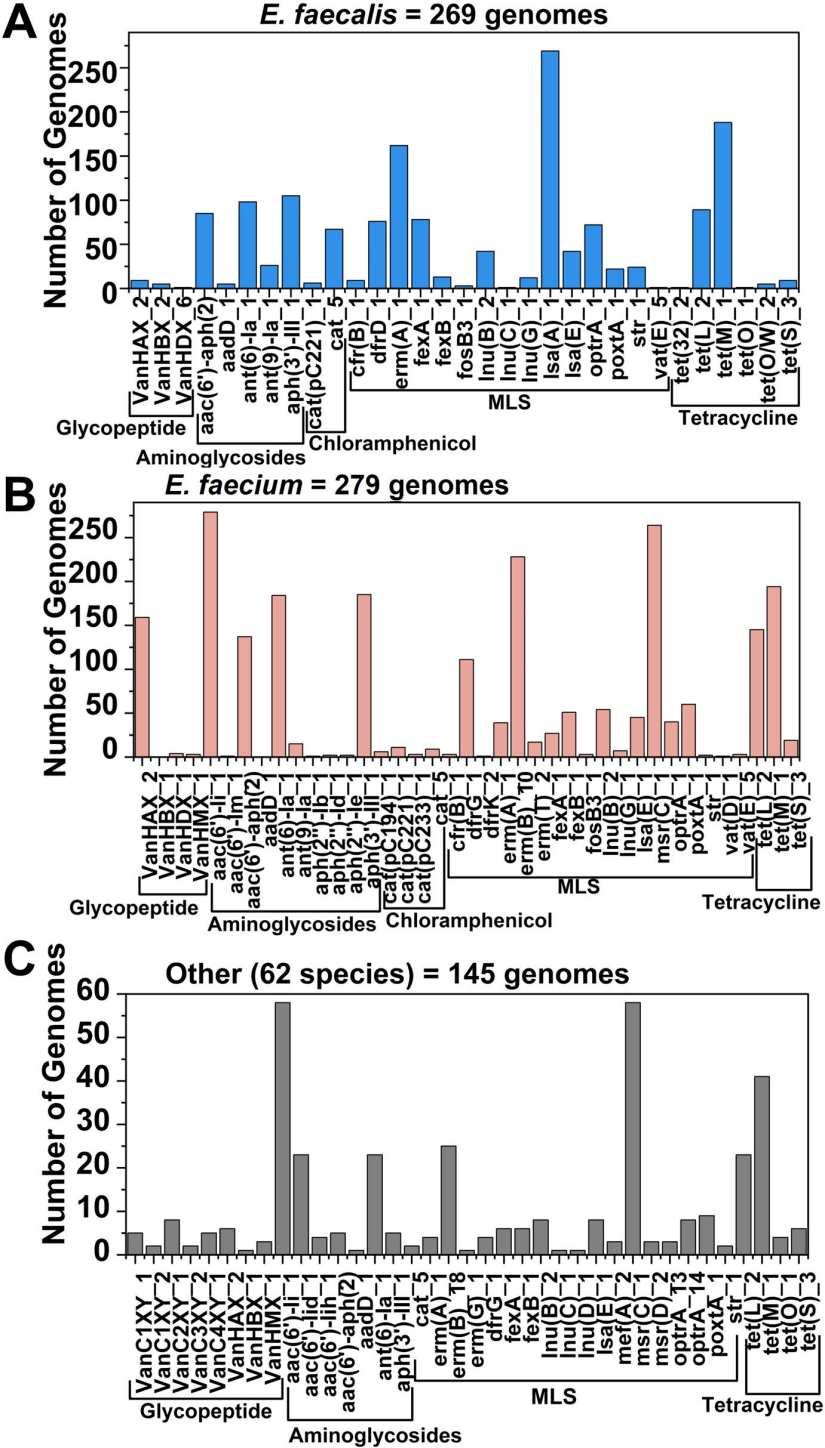
The prevalence of antibiotic resistance genes in 702 complete genome sequences of genus *Enterococcus* with NCBI database annotations. Antibiotic resistance gene prevalence in the genomes of *E. faecalis* (**A**), *E. faecium* (**B**), and 62 other species (**C**).

Using genome-wide analysis, we found that the majority of the genomes of *E. faecium* contained genes predicted to provide resistance to antibiotics such as vancomycin, aminoglycosides, the macrolide–lincosamide–streptogramin B (MLS) group of antibiotics as well as tetracycline. In the genomes of *E. faecalis*, genes predicted to provide resistance to the MLS group of antibiotics and the resistance gene (*lsa*(A)) were found exclusively. More than half of the genomes of *E. faecalis* were found to contain a tetracycline resistance gene (*tetM*). The tetracycline resistance genes *tetM* and *tetL* were found to coexist in most genomes (Fig. 5A through C). Most tetracycline-resistant isolates carried the *tetM* gene, coding for a ribosomal protection protein, and four isolates additionally harbored the *tetL* gene, which codes for energy-dependent efflux protein. Those genes were also present in susceptible *Enterococcus* isolates (82). However, vancomycin resistance gene distribution was low in *E. faecalis*, although vancomycin resistance genes were prevalent in the genomes of *E. faecium* (Fig. 5B). Likewise, most genomes of *E. faecium* contain tetracycline resistance genes. When we compared the prevalence of vancomycin and tetracycline resistance genes, these were frequently found in the genomes of *E. faecium* and less often in *E. faecalis* and other species.

#### Antibiotic resistance gene distribution in species other than *E. faecalis* and *E. faecium*

**Fig 6:**
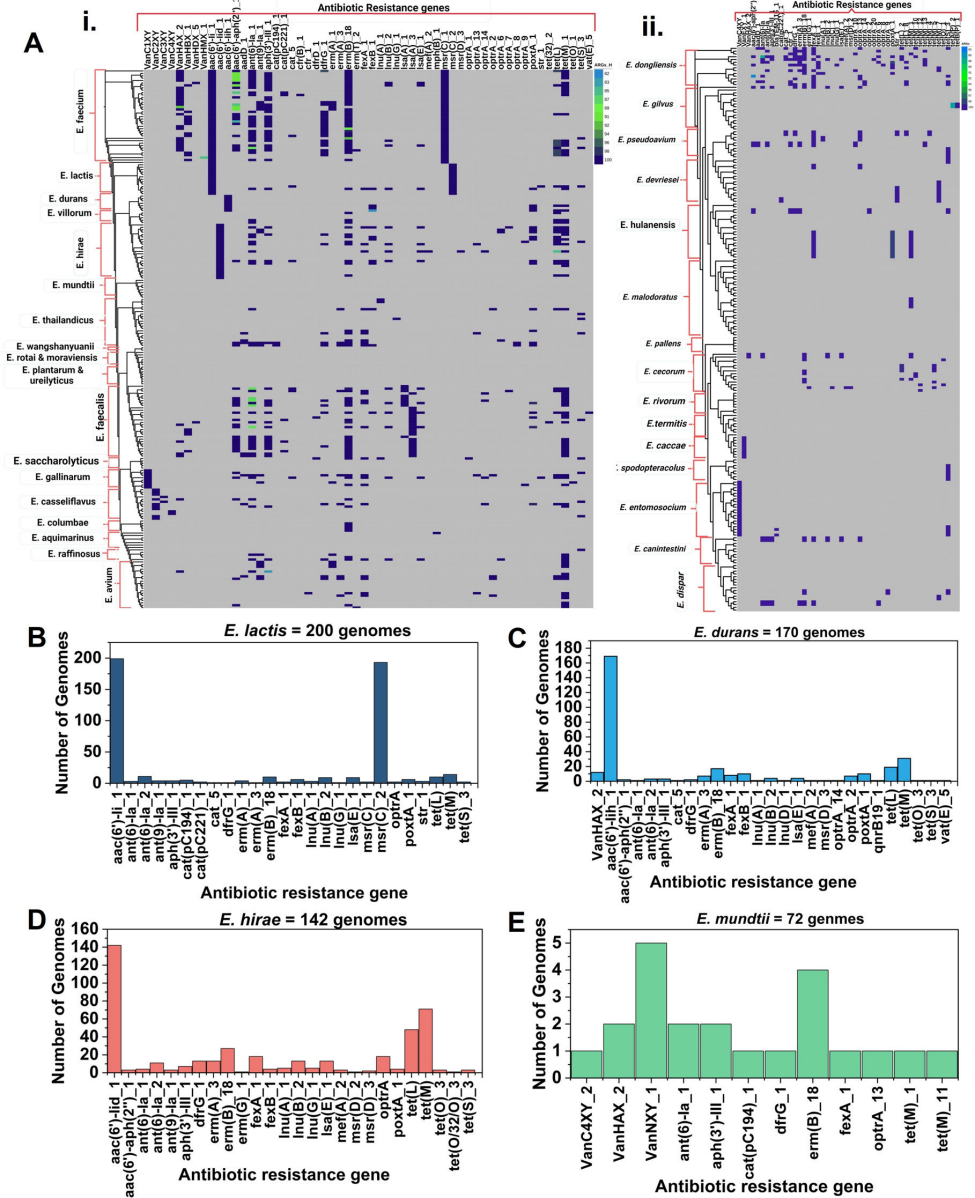
Prevalence of antibiotic resistance genes in species other than *E. faecalis* and *E. faecium*. The antibiotic resistance gene distribution in 427 whole-genome sequences of 34 different species downloaded from the NCBI database (**A**). Antibiotic resistance gene prevalence in the genomes of *E. lactis* (**B**), *E. durans* (**C**), E. *hirae* (**D**), and *E. mundtii* (**F**).

In the 427 genomes representing other species than *E. faecalis* and *E. faecium*, we found vancomycin resistance genes to be prevalent in the genomes of *E. gallinarum*, *E. casseliflavus*, *E. dongliesis*, *E. pseudoavium*,* E*. *entomosocium*, and *E. casseliflavus*. It has been reported that the vancomycin resistance in the abovementioned species is due to the *vanC* gene (84). Most species, however, did not contain any antibiotic resistance genes, including *E. rotai*, *E. moraviensis*, *E. plantarum*, *E. saccharolyticus*, *E. ureilyticus*, *E. pallens*, *E. rivorum*, and *E. termitis* (Fig. 6A, i and ii). From all detected antibiotic resistance genes *aac(6’)-li_1*, encoding a class of aminoglycosides and *msr(C)*, encoding an MLS class antibiotic resistance, could only be found in the genomes of *E. faecium* and *E. lactis* (Fig. 6A, i). It has been reported that among resistance genes, the aminoglycoside resistance gene *aac (8)-Ii* and genes encoding resistance to macrolides and streptogramins (*msrA/B*, *msr(C*)) and tetracycline (*tetM*) occur most frequently. The MLS group antibiotic resistance gene, *msr(C*), has also been found in both erythromycin-resistant and sensitive enterococci (82).

In the genomes of *E. lactis*, two resistance genes, an aminoglycoside resistance gene [*aac(6')-Ii_1*] and a macrolide resistance gene [*msr(C)_2*] were found in all 200 analyzed genomes. However, only a very limited number of genomes contained other antibiotic resistance genes. None of the genomes carried vancomycin resistance genes and tetracycline resistance genes were identified in just four out of the 200 genomes (Fig. 6B). Similarly, the genomes of *E. durans* also contained an aminoglycoside resistance gene [*aac(6’)-lih_1*] exclusively while a limited number of genomes contained other antibiotic resistance genes (Fig. 6C). For *E. hirae*, similar results were observed in that there was one aminoglycoside resistance gene [*aac(6’)-lid_1*] in most genomes besides other resistance genes found in a smaller number of the genomes (Fig. 6D). For *E. mundtii*, only 12 of 72 analyzed genomes contained antibiotic resistance genes (Fig. 6E). In our previous study, we found that the genomes of certain isolates carried genes for resistance to aminoglycosides, erythromycin, macrolides, and streptogramin B, yet they remained phenotypically sensitive (4). It is also known that several food-grade LAB harbor different genes that cause antimicrobial resistance(AMR); *Lactobacillus paracasei* BCRC-16100 and *L. paracasei* ZFM54 contain AMR genes particularly against vancomycin and tetracycline, which were found to be transposable (85).

The EFSA guideline published in 2012 emphasizes the importance of monitoring and controlling antibiotic resistance in probiotics and other microbial strains used in food and feed. In particular, the guideline specifies that *Enterococcus* species, commonly utilized in probiotics and starter cultures, must demonstrate susceptibility to the antibiotic ampicillin, defined by a Minimum Inhibitory Concentration of ≤2 mg/L (52).

Ampicillin is a crucial antibiotic for treating *Enterococcus* infections. Resistance to it poses significant risks, especially for vulnerable populations. To mitigate such risks, EFSA emphasizes the need to prevent the introduction and spread of microorganisms carrying antibiotic-resistance genes into the food chain, as this could ultimately affect humans and animals (35, 37, 52, 86). Enterococci-based probiotic strains intended for use in food production or as dietary supplements should therefore not harbor transferable resistance genes. Our thorough investigation has shown that despite the occurrence of culprits in the genus, there is much unrealized potential.

The capacity for horizontal transfer has often been used as an argument against using enterococci in food fermentations (87). Such a capacity is not uniquely associated with enterococci, and studies have revealed that LAB in general are the result of extensive horizontal transfer events (88). Some enterococci even harbor genes encoding putative CRISPR-Cas defense mechanisms that are considered barriers to horizontal gene transfer (89). For example, *E. durans*, a minor gut flora component with potential probiotic properties, lacks virulence genes and carries CRISPR arrays flanked by Cas genes, including *cas9*, *cas1*, *cas2*, and *csn2* (4).

### Conclusion

*Enterococci* have a great untapped industrial potential that has remained unexplored due to concerns about their safety. By scrutinizing a large number of genome sequences stored in the NCBI database, we found that antibiotic resistance and virulence genes were mainly present in the two *Enterococcus* species that dominate the gut microbiota, for example, *E. faecalis* and *E. faecium*. Apart from *E. faecalis*, in other species, few virulence genes other than those associated with adherence could be identified. Adherence-related genes are commonly found in various probiotic and food-origin bacterial species. Resistance to vancomycin, tetracycline, and ampicillin poses a significant challenge when treating enterococcal infections, although such resistance is limited to certain species. We identified several species that lacked virulence and antibiotic resistance genes altogether. From the analysis, we conclude that the picture of the genus *Enterococcus* is predominantly negative. This emphasis neglects the existence of an abundance of species that do not harbor any potential virulence or antibiotic resistance genes, species which appear to mainly reside in other places than the animal gut. Therefore, we recommend that members of the genus could be considered for use in food fermentation, if deemed safe based on both genomic and physiologic characterization.

## SUPPLEMENTAL MATERIAL

Supplemental figures aem.00415-25-s0001.docx Figures S1 and S2.

Supplemental legends aem.00415-25-s0002.docx Legends for Tables S1 to S3.

Table S1 aem.00415-25-s0003.xlsx Similarity percentages of the detected virulence and antibiotic resistance genes for 702 complete genomes.

Table S2 aem.00415-25-s0004.xlsx Similarity percentages of the detected virulence genes in the genomes of *E. lactis, E. durans, E. hirae*, and *E. mundtii*.

Table S3 aem.00415-25-s0005.xlsx Similarity percentages of the detected antibiotic resistance genes in the genomes of *E. lactis, E. durans, E. hirae*, and *E. mundtii*.

ASM does not own the copyrights to Supplemental Material that may be linked to, or accessed through, an article. The authors have granted ASM a non-exclusive, world-wide license to publish the Supplemental Material files. Please contact the corresponding author directly for reuse.
