# Supplementary material for: Genome analysis reveals a biased distribution of virulence and antibiotic resistance genes in the genus Enterococcus and an abundance of safe species
Source: Appl Environ Microbiol. 2025 Apr 9;91(5):e00415-25. doi: 10.1128/aem.00415-25 (PMC12094018; doi:10.1128/aem.00415-25)
Supplement: Supplemental figures — Figures S1 and S2. [file aem.00415-25-s0001.docx]

**Genome Analysis Reveals a Biased Distribution of Virulence and Antibiotic Resistance Genes in the Genus *Enterococcus* and an Abundance of Safe Species**

Belay Tilahun Tadesse^1,3^, Shuangqing Zhao^1^, Liuyan Gu^1^, Carsten Jers^3^, Ivan Mijakovic^2,3^ and Christian Solem^1^*

^1^National Food Institute, Research Group for Microbial Biotechnology and Biorefining, Technical University of Denmark, Lyngby, Denmark

^2^Novo Nordisk Foundation Center for Biosustainability, Kongens Lyngby, Denmark,

^3^Systems and Synthetic Biology Division, Department of Biology and Biological Engineering, Chalmers University of Technology, Gothenburg, Sweden

***Corresponding author**: **Christian Solem**; e-mail: [chso@food.dtu.dk](mailto:chso@food.dtu.dk)


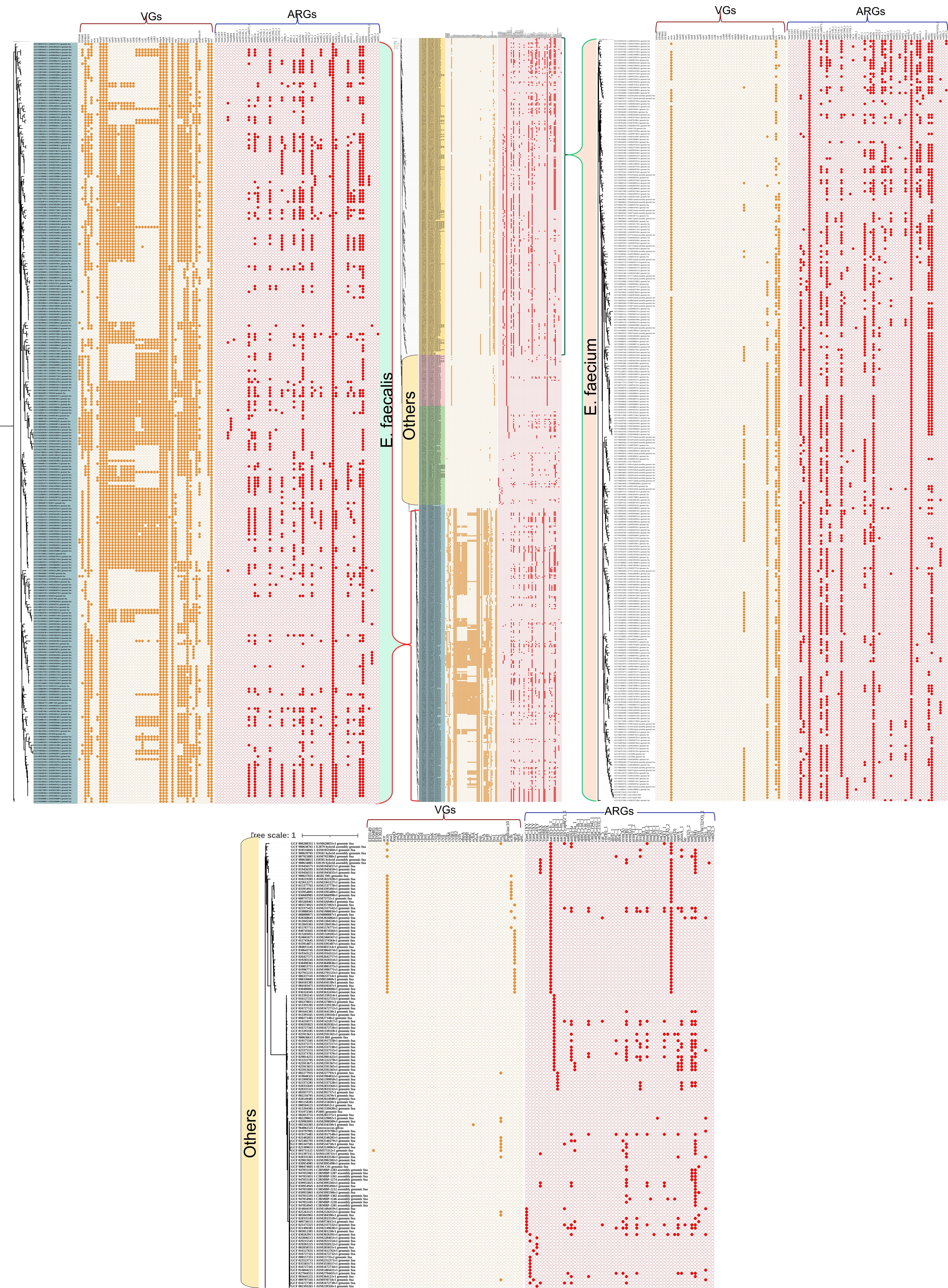


**Fig. S1** Distribution of virulence genes prevalence in the genomes of *E. faecalis*, *E. faecium* and 62 other species. The shape filled with distinct colors indicates presence and the shape without a color filled indicates absence.


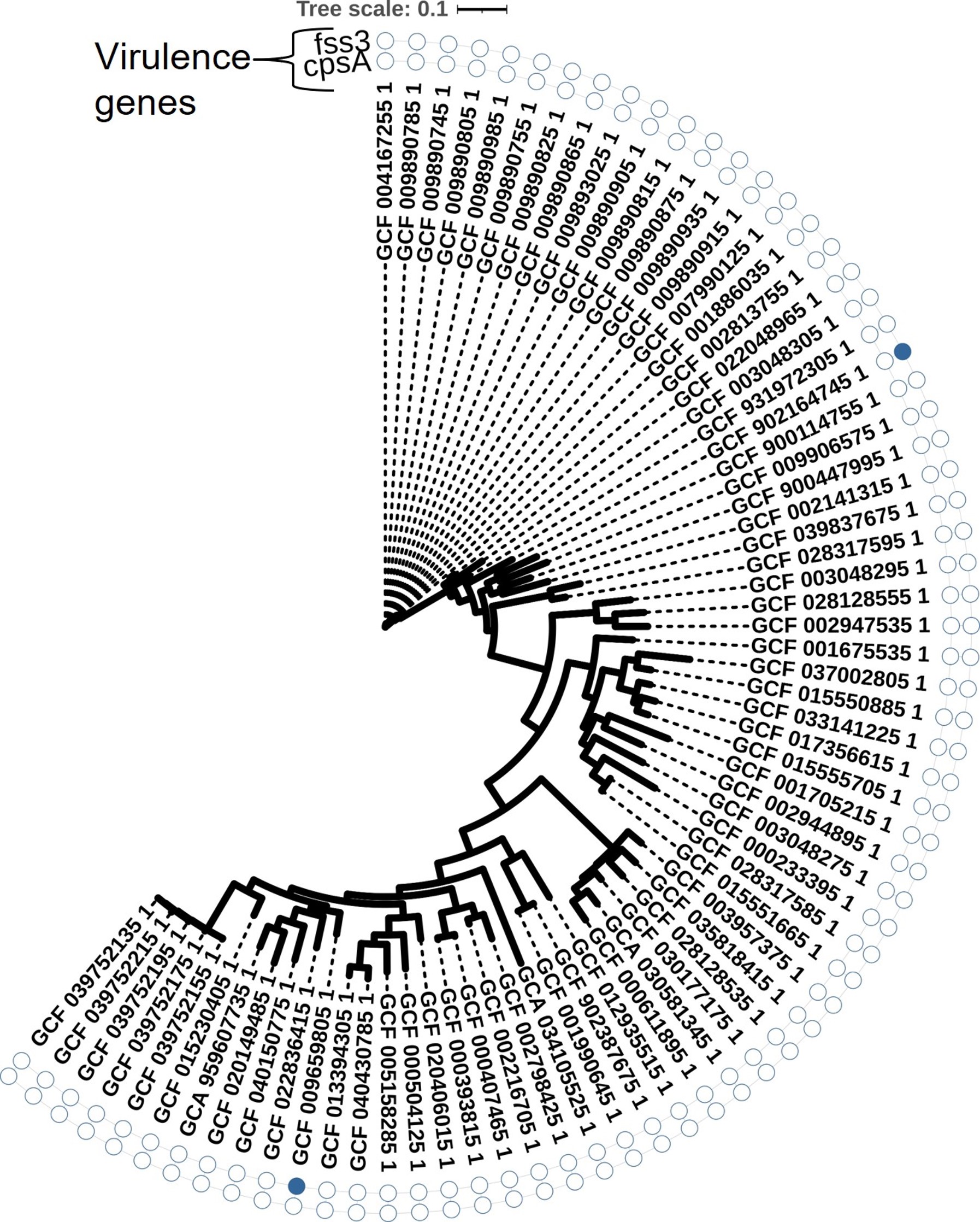


**Fig. S2** Presence of virulence genes in the genomes of *E. mundtii*, the shape filled with distinct colors indicates presence and the shape without a color filled indicates absence.
