# Supplementary material for: Genome analysis reveals a biased distribution of virulence and antibiotic resistance genes in the genus Enterococcus and an abundance of safe species
Source: Appl Environ Microbiol. 2025 Apr 9;91(5):e00415-25. doi: 10.1128/aem.00415-25 (PMC12094018; doi:10.1128/aem.00415-25)
Supplement: Supplemental legends — Legends for Tables S1 to S3. [file aem.00415-25-s0002.docx]

**Genome Analysis Reveals a Biased Distribution of Virulence and Antibiotic Resistance Genes in the Genus *Enterococcus* and an Abundance of Safe Species**

Belay Tilahun Tadesse^1,3^, Shuangqing Zhao^1^, Liuyan Gu^1^, Carsten Jers^3^, Ivan Mijakovic^2,3^ and Christian Solem^1^*

^1^National Food Institute, Research Group for Microbial Biotechnology and Biorefining, Technical University of Denmark, Lyngby, Denmark

^2^Novo Nordisk Foundation Center for Biosustainability, Kongens Lyngby, Denmark,

^3^Systems and Synthetic Biology Division, Department of Biology and Biological Engineering, Chalmers University of Technology, Gothenburg, Sweden

***Corresponding author**: **Christian Solem**; e-mail: [chso@food.dtu.dk](mailto:chso@food.dtu.dk)

**Supplementary table legend**

**Supplementary table S1.** The percentage of similarity of the detected virulence and antibiotic resistance genes for 702 complete genomes. arranged in two excel cells the first cell virulence gene the second one antibiotic resistance gene.

**Supplementary table S2.** The percentage of similarity of the detected virulence genes genomes arranged in excel of *E. durans*, *E. lactis* and *E. hiare*.

**Supplementary table S3.** The percentage of similarity of the detected antibiotic resistance genes genomes arranged in excel of *E. durans*, *E. lactis* *E. hiare* and *E. mundtii*.
